# Influence of Different Pigmentations and Accelerated Aging on the Hardness and Tear Strength of the A-2186 and MDX4-4210 Silicones

**DOI:** 10.1155/2020/8492091

**Published:** 2020-08-20

**Authors:** Estefânia Marrega Malavazi, Daniela Micheline dos Santos, Clóvis Lamartine de Moraes Melo Neto, Fernanda Pereira de Caxias, Emily Vivianne Freitas da Silva, Lisiane Cristina Bannwart, Aldiéris Alves Pesqueira, André Luiz de Melo Moreno, André Pinheiro de Magalhães Bertoz, Marcelo Coelho Goiato

**Affiliations:** ^1^Department of Dental Materials and Prosthodontics, São Paulo State University (UNESP), School of Dentistry, Aracatuba, São Paulo, Brazil; ^2^Oral Oncology Center, São Paulo State University (UNESP), School of Dentistry, Araçatuba, São Paulo, Brazil; ^3^Brazilian Institute of Northern Education (IBEN), Manaus, Amazonas, Brazil; ^4^Department of Pediatric and Social Dentistry, São Paulo State University (UNESP), School of Dentistry, Araçatuba, São Paulo, Brazil

## Abstract

**Objective:**

To evaluate the influence of different pigmentations and accelerated aging on the hardness and tear strength of the A-2186 and MDX4-4210 silicones.

**Materials and Methods:**

The samples A-2186 and MDX4-4210 were manufactured without and with pigmentations (black, bronze, and pink). For the Shore A hardness test, 80 samples of each silicone were fabricated, and for the tear strength test, 320 samples of each silicone were fabricated. Eight groups were created for each test (*n* = 10). These tests were performed before and after 252, 504, and 1008 hours of aging. Three-way repeated-measures analysis of variance and the Tukey test were performed (*α* = 0.05).

**Results:**

The A-2186 silicone showed higher hardness and tear strength when compared with the MDX4-4210 silicone (*p* < 0.05), except in the hardness of the A-2186 and MDX4-4210 groups without pigmentation after 1008 hours (*p* > 0.05). All hardness values were between 25 and 35 units, regardless of the silicone type, period, and pigmentation (or no pigmentation). In most situations, the hardness of silicones used increased after 252 hours (*p* < 0.05). The nonpigmented MDX4-4210 group and all A-2186 groups showed an increase in tear strength after 252 hours (*p* < 0.05). For the nonpigmented MDX4-4210 group, from 252 to 1008 hours, there was no change in tear strength (*p* > 0.05). All pigmented MDX4-4210 groups showed no change in tear strength from 0 (initial) to 1008 hours of aging (*p* > 0.05). In all A-2186 groups, from 252 to 504 hours, there was a reduction in tear strength (*p* < 0.05), and from 504 to 1008 hours, there was an increase in tear strength (*p* < 0.05), except in the bronze A-2186 group (*p* > 0.05).

**Conclusion:**

In most situations, the A-2186 silicone showed significantly higher values of hardness and tear strength than the MDX4-4210 silicone. All hardness values were considered clinically acceptable. Accelerated aging could increase, decrease, or not significantly change the hardness and tear strength of the silicones used. The results of hardness and tear strength suggest that MDX4-4210 was more influenced by the presence of pigmentation after aging.

## 1. Introduction

Facial deformities can be caused by congenital malformation, oncologic surgery, or trauma [1–5] and are embarrassing for patients [1, 6]. Maxillofacial prostheses are very important for patients with facial deformities, especially when facial reconstruction with plastic surgery is not possible [1, 3, 4, 6]. These prostheses also protect the affected facial region [5] and can improve the self-esteem, aesthetics, and quality of life of the patient [1, 2, 5, 6].

Silicone was introduced in maxillofacial prostheses field during the 1960s [2] and later became the material of choice for the manufacture of these prostheses [2–10]. According to Hatamleh et al. [3], silicones have many desirable properties including biocompatibility, ease of manipulation, low viscosity, and patient accommodation properties (i.e., nontoxic, easily cleansable, lightweight, and compatible with adhesives) [3]. Despite these advantages, the clinical durability of silicone prostheses can vary only from 3 [5] to 12 months [1].

Ultraviolet rays, temperature fluctuations, and humidity are conditions that can degrade the mechanical properties of silicones (e.g., tear strength and hardness) [1, 4, 5, 7, 8]. One of the most important properties for silicone maxillofacial prostheses is the tear strength [9, 10]. According to Aziz et al. [9], it is important that a silicone with a high resistance to tearing is used to construct a maxillofacial prosthesis [9]. This is important because the margins of a silicone prosthesis are usually glued to the patient's face using a medical adhesive [3, 9, 10]. Thus, the thin margins of this type of prosthesis are susceptible to tearing as the prosthesis is removed from the facial tissue [3, 9, 10]. Another important property for a silicone is the surface hardness. According to Hatamleh et al. [3], the hardness of silicone elastomers is controlled by the surface characteristics of the polymer network and by the density of cross-links [3]. The hardness of a silicone determines its flexibility and allows the prosthesis to mimic the skin texture, promoting greater comfort for the patient [5, 8, 9].

Few studies have compared the mechanical properties between MDX4-4210 and A-2186 silicones [7, 11–13]. Dootz et al. [7], Haug et al. [11], and Sanchez et al. [12] performed this type of comparison; however, these authors did not incorporate pigments in these silicones [7, 11, 12]. Only the study performed by Goiato et al. [13] compared the tear strength between these silicones with different pigmentations [13]. However, Goiato et al. [13] did not evaluate different aging periods and the Shore A hardness [13]. According to Abdullaha and Abdul-Ameerb [14], the mechanical and physical properties of silicones are reported by manufacturers only without pigments and opacifiers, and this does not represent a real clinical situation for a silicone prosthesis [14] because clinically this type of prosthesis is pigmented. It is important to emphasize that the mechanical properties of a silicone elastomer can be influenced due to the presence of an intrinsic pigmentation [5]. Therefore, this study aimed to evaluate the influence of different pigmentations and accelerated aging on the hardness and tear strength of the A-2186 and MDX4-4210 silicones.

## 2. Materials and Methods

The MDX4-4210 (Dow Corning Corporation Medical Products, USA) and A-2186 (Factor II, USA) silicones and three different intrinsic pigmentations were selected. Bronze (Functional Intrinsic II-215, Factor II, USA) and black (Functional Intrinsic II-205, Factor II, USA) pigments were specific for characterization of prostheses. In addition, in this study, a new medium pink pigmentation (Orbital Colors, Brazil) was tested [13, 15]. The pink pigmentation was created by mixing the yellow, red, and black pigments (Orbital Colors) with a white opacifier (TiO_2_) (Orbital Colors) [13, 15]. In this study, the black pigment (Functional Intrinsic II-205, Factor II) generated the black pigmentation; the bronze pigment (Functional Intrinsic II-215) generated the bronze pigmentation; and the mixture between the TiO_2_ opacifier and the yellow, red, and black pigments (Orbital Colors) generated the pink pigmentation.

### 2.1. Creation of Groups

Eight groups were created for each test (Shore A hardness and the tear strength) (*n* = 10). The groups were manufactured according to the silicone type (A-2186 and MDX4-4210), period (initial and after 252, 504, and 1008 hours of aging), and pigmentation used (bronze, black, and pink) or not (silicone without pigmentation). Group 1: A-2186 without pigmentation; group 2: A-2186 + bronze pigmentation; group 3: A-2186 + black pigmentation; group 4: A-2186 + pink pigmentation; group 5: MDX4-4210 without pigmentation; group 6: MDX4-4210 + bronze pigmentation; group 7: MDX4-4210 + black pigmentation; and group 8: MDX4-4210 + pink pigmentation. Eighty samples were manufactured for the hardness test, and 320 samples were manufactured for the tear strength test. The number of samples for the tear strength test was greater when compared with the hardness test because in each evaluated period, the samples for the tear strength test were lost. For the hardness test, the samples were not lost in each evaluated period.

### 2.2. Samples Fabrication

Silicones, pigments, and the opacifier were weighed on a digital analytical balance (Adventurer, Ohaus Corporation, USA). Each Factor II pigment (bronze and black) corresponded to 0.2% of the weight of its respective silicone [1, 13, 15, 16]. For the pink pigmentation (Orbital Colors), the pigments that constituted it corresponded to 0.122% (yellow), 0.006% (black), and 0.03% (red) of the weight of its respective silicone. In addition, the opacifier (TiO_2_) corresponded to 0.6% of the weight of its respective silicone [13, 15]. In this study, all tested pigments had an organic origin, and the opacifier (TiO_2_) had a mineral origin [13, 15].

Each silicone was handled according to the recommendations of its respective manufacturer at room temperature (23 ± 2°C) [1, 2, 5, 13–16] and under a relative humidity of 50 ± 10% [14, 16]. For each test (hardness and tear strength), a metallic matrix with specific dimensions for the manufacture of samples was used. After the manipulation, the silicone was inserted into the matrix, and the thickness was regularized with the aid of a metal spatula. Subsequently, the samples contained in the matrix were exposed to the environment for 72 hours (27 ± 2°C) to complete polymerization of the material [1, 2, 5, 6, 8, 13, 15, 16]. Posteriorly, the samples were carefully separated from the matrix [5]. Subsequently, the samples were stored in a dark chamber at room temperature (23 ± 2°C) and under a relative humidity of 50 ± 5% until the beginning of the tests, aiming to avoid exposure to ambient light [3, 13]. All samples were manufactured by the same operator.

### 2.3. Shore A Hardness

**Figure 1:**
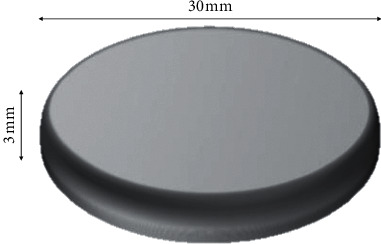
Dimensions of the samples for the Shore A hardness test.

Samples were manufactured with standardized dimensions (30 mm in diameter × 3 mm in height) [6, 17, 18] (Figure 1). The hardness test was performed using a digital durometer (GSD 709 Teclock, Japan), according to the American Society for Testing and Materials (ASTM) Designation D2240 [3–6, 8, 11, 14, 19]. The measurement was established between 0 and 100 Shore A units, with ±1% of tolerance [6, 19]. The hardness values were expressed in Shore A units [5, 18, 19]. Each sample was positioned on the stand of the hardness meter at a distance of ±2 mm from the penetration tip of the appliance [19]. The needle penetrated the samples at a load of 10 N for 15 seconds [8, 19]. Three measurements were performed on each sample in each period [6, 18, 19]. Subsequently, for each sample, a mean of the 3 measurements was obtained.

### 2.4. Tear Strength

**Figure 2:**
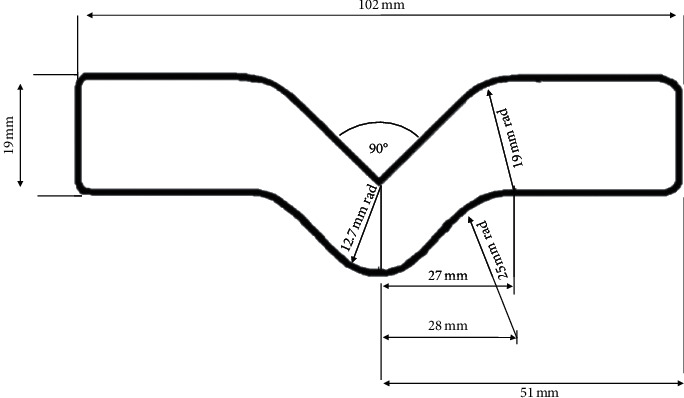
Dimensions of the samples based on ASTM D-624 (type C).

The tear strength test was performed according to the ASTM D-624 (type C) [4, 10, 11, 13, 14]. All samples were tested using a universal testing machine (EMIC, Instron, Brazil). The manufactured samples had standardized dimensions (Figure 2) [11, 13]. The thickness of all samples was 3 mm. Samples were stretched at a rate of 500 mm/min [4, 10, 11, 13]. The formula *T*=(*F*/*D*) was used, with *F* being the maximum force (Newton) required to break the sample and *D* being the thickness (mm) of the sample [4, 10, 11, 13, 14]. The results were obtained in Newton/mm (N/mm).

Hardness and tear strength tests were performed by the same operator.

### 2.5. Accelerated Aging

Accelerated aging was performed according to the ASTM Designation G53-96 [20]. Samples were placed in a chamber for nonmetallic samples (Equilam, Brazil) and subjected to alternating periods of ultraviolet B light (UVB-313 lamps, 40 Watts, Equilam) and distilled water condensation saturated with oxygen under conditions of heat and 100% humidity [1, 5, 8, 13, 15, 16]. Each aging cycle lasted 12 hours [1, 5, 8, 13, 15, 16]. In the first 8 hours, the temperature was maintained at 60 ± 3°C, and the ultraviolet B light was imputed onto the samples [1, 5, 8, 13, 15, 16]. In the last 4 hours, the temperature was maintained at 45 ± 3°C, and a condensation period occurred without light [1, 5, 8, 13, 15, 16]. The aging was performed for a total of 1008 hours, and the deterioration caused by rain, dew, and ultraviolet light from the sun was simulated [1, 5, 8, 16]. Hardness and tear strength tests were performed initially and after 252, 504, and 1008 hours of accelerated aging [1, 2, 6, 8, 16].

### 2.6. Data Analysis

All data were analyzed using the Statistical Package for Social Sciences 20.0 (IBM Corp., USA). Data were submitted to three-way repeated-measures analysis of variance (ANOVA) (factors: pigmentation, silicone, and period) and the Tukey test, with a level of significance of 5%.

## 3. Results

The interaction between pigmentation, silicone, and period interfered with the results of hardness (*p* < 0.001) and tear strength (*p* = 0.045). Tables 1to 4 show the mean and standard deviation (SD) of the hardness and tear strength values of all groups.

The hardness values increased significantly after 252 hours of aging in all MDX4-4210 and A-2186 groups (*p* < 0.05), except in the MDX4-4210 group without pigmentation (Table 1). In all MDX4-4210 and A-2186 groups, the aging from 252 to 504 hours did not significantly change the hardness values (*p* > 0.05), except in the MDX4-4210 group without pigmentation that showed a higher hardness value (*p* < 0.05) (Table 1). In all MDX4-4210 and A-2186 groups, the aging from 504 to 1008 hours did not significantly change the hardness values (*p* > 0.05), except in the black A-2186 group that showed a significant increase in the hardness value (*p* < 0.05) and in the A-2186 group without pigmentation that showed a significant reduction in the hardness value (*p* < 0.05) (Table 1).

For the hardness test, when the pigmented groups were compared with the group without pigmentation (Table 1) for each silicone and in the same period, it was possible to observe significantly lower values in the pigmented groups (*p* < 0.05): pink A-2186 and bronze MDX4-4210 (initial); bronze MDX4-4210 (252 hours of aging); pink MDX4-4210 and bronze MDX4-4210 (504 hours of aging); and pink MDX4-4210 and bronze MDX4-4210 (1008 hours of aging) (Table 1). After 1008 hours of aging, the black A-2186 group and the pink A-2186 group showed higher hardness values when compared with the A-2186 group without pigmentation (*p* < 0.05) (Table 1).

After 252 hours of aging, there was a significant increase in tear strength values in the MDX4-4210 group without pigmentation and also in all A-2186 groups (without pigmentation, black, bronze, and pink) (*p* < 0.05) (Table 2). After 504 hours of aging, all groups A-2186 showed a significant reduction in tear strength values when compared with the period of 252 hours of aging (*p* < 0.05) (Table 2). After 1008 hours of aging, all A-2186 groups showed a significant increase in tear strength values when compared with the period of 504 hours of aging (*p* < 0.05), except in the A-2186 bronze group (*p* > 0.05) (Table 2). The MDX4-4210 group without pigmentation did not show a significant change in tear strength values from 252 to 504 hours of aging and from 504 to 1008 hours of aging (*p* > 0.05) (Table 2). All pigmented MDX4-4210 groups did not show a significant change in tear strength from 0 (initial) to 1008 hours of aging, regardless of the periods compared (*p* > 0.05) (Table 2).

For the tear strength test, when the pigmented groups were compared with the group without pigmentation (Table 2) for each silicone and in the same period, it was possible to observe that initially, for the MDX4-4210 and A-2186 silicones, the group without pigmentation was statistically similar to the pigmented groups (*p* > 0.05). This situation was similar to the results of the A-2186 silicone after 252 and 504 hours of aging (*p* > 0.05) (Table 2). After 1008 hours of aging, the tear strength value of the A-2186 group without pigmentation was statistically higher than the tear strength value of the bronze A-2186 group (*p* < 0.05) (Table 2). In the MDX4-4210 groups, the group without pigmentation showed a statistically higher value of tear strength than all pigmented groups after 252 and 504 hours of aging (*p* < 0.05). After 1008 hours of aging, only the pink MDX4-4210 group showed a statistically lower value of tear strength when compared with the MDX4-4210 group without pigmentation (*p* < 0.05) (Table 2).

When the MDX4-4210 silicone was compared with the A-2186 silicone in each period of aging and with the same pigmentation (or no pigmentation), the hardness values were higher for the A-2186 silicone (*p* < 0.05), except in the MDX4-4210 and A-2186 groups without pigmentation after 1008 hours (*p* > 0.05) (Table 3). When the same comparison was performed for the tear strength values, the results were statistically higher for the A-2186 silicone in all situations (*p* < 0.05) (Table 4).

## 4. Discussion

After 252 hours of accelerated aging, the hardness values of all A-2186 and MDX4-4210 groups increased statistically (*p* < 0.05), except in the MDX4-4210 group without pigmentation (*p* > 0.05) (Table 1). In addition, after 252 hours of aging, the tear strength values of the MDX4-4210 group without pigmentation and also of all A-2186 groups (without pigmentation, black, bronze, and pink) increased (*p* < 0.05) (Table 2). These results may have occurred due to the continuous polymerization of the MDX4-4210 and A-2186 silicones associated with exposure to ultraviolet B rays [5, 6, 13]. It is important to note that after 252 hours of aging, all pigmented MDX4-4210 groups did not show a significant increase in tear strength values (*p* > 0.05) (Table 2). Probably, the pigments and opacifier used in this study may have hindered the intertwining of the polymer chains of the MDX4-4210 silicone, reducing its polymerization rate [5]. This suggests that the MDX4-4210 silicone was more influenced by pigmentations (black, bronze, and pink) when compared with the A-2186 silicone after aging.

Based on the results of this study, it is possible to suggest that the highest rates of polymerization occurred in the period of 252 hours of aging, for most samples of the hardness and tear strength tests. This can be attributed to the fact that, after 504 and 1008 hours of aging, the hardness values of all MDX4-4210 and A-2186 samples did not increase statistically significantly when compared with the hardness values of the period of 252 hours of aging (*p* > 0.05), except in the MDX4-4210 group without pigmentation (after 504 and after 1008 hours) and in the bronze MDX4-4210 group (after 1008 hours) (*p* < 0.05) (Table 1). This situation was similar to the results of the tear strength test (Table 2). After 504 and 1008 hours of aging, in all A-2186 groups and in the nonpigmented MDX4-4210 group, the tear strength values did not increase statistically significantly when compared with the tear strength values of the period of 252 hours of aging (*p* > 0.05) (Table 2).

In this study, it is interesting to note that in all A-2186 groups, there was a significant reduction in tear strength values from 252 to 504 hours of aging (*p* < 0.05), and subsequently, there was a significant increase in tear strength values from 504 to 1008 hours of aging (*p* < 0.05), except in the bronze A-2186 group (*p* > 0.05) (Table 2). These results are probably related to a silicone degradation. In addition, based on these results, a degradation process in all groups MDX4-4210 may also have occurred from 252 to 1008 hours of aging. Despite the absence of a statistically significant difference in tear strength values between 252, 504, and 1008 hours of aging in all MDX4-4210 groups (*p* > 0.05), it is possible to verify that numerically, from 252 to 504 hours of aging and from 504 to 1008 hours of aging, all MDX4-4210 groups had a reduction in tear strength values and subsequently, an increase in tear strength values (*p* > 0.05) (Table 2). Therefore, probably, although a degradation in all MDX4-4210 groups from 252 to 1008 hours of aging has occurred, this degradation was insufficient to cause a significant change in tear strength values. It is important to mention that the A-2186 silicone probably has a higher filler loading and molecular weight of the dimethylsiloxane polymer when compared with the MDX4-4210 silicone [21]. Thus, these possible different characteristics between these two silicones may have influenced their level of degradation, based on the property of tear strength. In addition, significant changes in the hardness of the MDX4-4210 and A-2186 silicones, from 252 to 1008 hours of aging, can also be the result of a degradation of these elastomers (Table 1).

In this study, when the pigmented groups were compared with the group without pigmentation, based on the same silicone and period, it was possible to observe that a pigmentation can generate, for example, a significant reduction in the hardness values of the MDX4-4210 and A-2186 silicones (mainly for the MDX4-4210 silicone) (Table 1). Therefore, based on the Shore A hardness results, the MDX4-4210 silicone was more influenced by the presence of a pigmentation when compared with the A-2186 silicone (Table 1). In addition, as previously reported, accelerated aging could significantly change the hardness values of the silicones used (Table 1). Despite these situations, according to some authors, the ideal Shore A hardness values of medical silicones to simulate the texture and flexibility of human skin should be between 25 and 35 units [4–6, 18, 19]. Therefore, all hardness values in this study were clinically acceptable, regardless of the period evaluated, silicone used, and the presence (or absence) of pigmentation (Table 1). For the hardness property, this situation shows the excellent quality of the A-2186 and MDX4-4210 silicones. Therefore, this may be one of the reasons why these silicones are widely used in the manufacture of maxillofacial prostheses [8, 13, 15]. In addition, in this study, it is interesting to note that the hardness values were not directly related to the tear strength values (Tables 1 and 2) [5]. Therefore, for example, a statistically significant increase in hardness values did not necessarily represent a statistically significant increase in tear strength values.

For the tear strength test, when the pigmented groups were compared with the group without pigmentation (Table 2) for each silicone and in the same period, it was possible to observe that initially there were no significant statistical differences, for the MDX4-4210 and A-2186 silicones (*p* > 0.05). This situation also occurred for the A-2186 silicone after 252 and 504 hours of aging (*p* > 0.05). On the other hand, the MDX4-4210 group without pigmentation showed a statistically higher value of tear strength when compared with all pigmented MDX4-4210 groups (bronze, black, and pink) after 252 and 504 hours of aging (*p* < 0.05). Probably as previously reported, the pigments and opacifier used in this study may have hindered the intertwining of the polymer chains of the MDX4-4210 silicone, reducing its polymerization rate [5]. Therefore, the tear strength property of the MDX4-4210 silicone was affected with the presence of the black, pink, and bronze pigmentations after aging.

For the tear strength test, after 1008 hours of aging, when the pigmented groups were compared with the group without pigmentation (Table 2) for each silicone and in the same period, it was possible to observe that only the bronze A-2186 group showed a significantly lower tear strength value when compared with the A-2186 group without pigmentation (*p* < 0.05) (Table 2). For the MDX4-4210 silicone, only the pink MDX4-4210 group showed a significantly lower tear strength value when compared with the MDX4-4210 group without pigmentation (*p* < 0.05) (Table 2). These results show differences when compared with the results patterns of the previous aging periods (after 252 and 504 hours of aging), for the MDX4-4210 and A-2186 silicones. Therefore, the results obtained after 1008 hours of aging are probably more related to the fact of the extreme aging of the silicones used than to the fact of the presence of a pigmentation. According to Goiato et al. [6] and Goiato et al. [13], the period of 1008 hours of accelerated aging corresponds to 1 year of constant use of a silicone prosthesis by a patient [6, 13]. In addition, many studies report that a silicone prosthesis has a maximum durability of one year [1, 8, 15, 18].

Based on the previous paragraphs discussed, for the hardness and tear strength tests, in most situations, the period of aging from 0 (initial) to 252 hours was probably more related to the polymerization process of the MDX4-4210 and A-2186 silicones. Evaluating the aging periods from 252 to 504 hours and from 504 to 1008 hours, it is possible to suggest that these aging periods were more related to the degradation of the silicones used. Tetteh et al. [22] can explain a situation of degradation of a silicone [22]. Tetteh et al. reported that the weathering may induce changes in physical, mechanical, and chemical characteristics of a polymer (e.g., silicone elastomer) [22]. The degradation of a polymer due to weathering is a result of a photo-oxidative attack (a combined action of oxygen and sunlight) on the chemical structure of this material [22]. The photo-oxidative degradation causes an initial formation of free radicals, reaction of free radicals with oxygen, production of polymer oxy- and peroxy-radicals, and secondary polymer radicals, resulting in chain scission [22]. In addition, a reaction of different free radicals with each other can result in crosslinking [22]. It is also important to report that a crosslinking may occur due to the formation of bonds between existing monomers or bonds between chains [22]. Other authors can also explain a situation of degradation of a silicone [2, 5, 15]. According to some authors, when a polymer molecule absorbs the ultraviolet light, this energy causes instability in its molecular structure [2, 5, 15]. The energy excess can be transmitted by excitation from a molecule to another, allowing the first molecule to recover its stability [2, 5, 15]. Then, affected groups can return to their original state by releasing energy in form of longer wavelength, such as heat or visible light [2, 5, 15]. However, when this excess energy is released, a photochemical degradation occurs, contributing to the deterioration of the molecule [2, 5, 15].

When the MDX4-4210 silicone was compared with the A-2186 silicone based on the same period and pigmentation (or no pigmentation), it was possible to observe higher values of hardness and tear strength for the A-2186 silicone (*p* < 0.05) (Tables 3 and 4), except for the hardness test in the MDX4-4210 and A-2186 groups without pigmentation after 1008 hours (*p* > 0.05) (Table 3). This may possibly have occurred due to the higher filler loading and/or higher molecular weight of the dimethylsiloxane polymer from the A-2186 silicone compared with the MDX4-4210 silicone [21]. These higher tear strength values for the A-2186 silicone corroborate the studies performed by Dootz et al. [7], Haug et al. [11], Sanchez et al. [12], and Goiato et al. [13]. According to Sanchez et al. [12], higher values of tear strength of the A-2186 silicone compared with the MDX4-4210 silicone may clinically indicate higher longevity of a maxillofacial prosthesis [12]. Regarding the hardness of the silicones used, the higher hardness values for the A-2186 silicone observed in the present study (Table 3) do not corroborate the studies by Haug et al. [11] and Sanchez et al. [12]. In these studies [11, 12], the MDX4-4210 silicone showed higher hardness than the A-2186 silicone (*p* < 0.05) [11, 12]. Dootz et al. reported that there was no hardness significant difference between these silicones before aging (*p* > 0.05) [7]. However, the A-2186 silicone showed higher hardness than MDX4-4210 silicone after aging (*p* < 0.05) [7]. Therefore, the study by Dootz et al. [7] partially corroborated the present study.

## 5. Conclusions

- All hardness values were clinically acceptable, regardless of the period evaluated, silicone used, and the presence (or absence) of pigmentation.
- In most situations, the A-2186 silicone showed significantly higher values of hardness and tear strength when compared with the MDX4-4210 silicone.
- All A-2186 groups showed a significant increase in tear strength from 0 (initial) to 252 hours of aging; a significant reduction in tear strength from 252 to 504 hours of aging; and a significant increase in tear strength from 504 to 1008 hours of aging, except in the bronze A-2186 group.
- The nonpigmented MDX4-4210 group showed a significant increase in tear strength after 252 hours of aging; however, from 252 to 1008 hours of aging, there was no significant change in tear strength values. All pigmented MDX4-4210 groups did not show a significant change in tear strength after aging, regardless of the period.
- The results of hardness and tear strength suggest that the MDX4-4210 silicone was more influenced by the presence of pigmentation after aging.

## Figures and Tables

**Table 1:** Mean (Shore A units) ± standard deviation for the hardness test of the A-2186 and MDX4-4210 silicones, according to the period and pigmentation (or no pigmentation).

Silicone	Pigmentation	Period			
		Initial	252 hours	504 hours	1008 hours
A-2186	Without pigmentation	29.64 ± 1.56 Aa	31.46 ± 1.56 ABb	31.46 ± 0.78 Ab	30.16 ± 2.60 Ca
	Bronze	29.64 ± 2.34 Aa	30.94 ± 1.56 Bb	31.72 ± 1.04 Ab	30.94 ± 0.26 Cb
	Black	30.94 ± 1.04 Aa	32.76 ± 1.04 Abc	31.98 ± 1.30 Ab	33.54 ± 1.30 Ac
	Pink	28.08 ± 2.08 Ba	31.20 ± 1.30 Bb	31.98 ± 1.04 Ab	32.24 ± 0.78 Bb

MDX4-4210	Without pigmentation	27.30 ± 1.82 Aa	28.34 ± 2.08 ABa	29.64 ± 1.30 Ab	30.16 ± 1.04 Ab
	Bronze	25.48 ± 1.04 Ba	26.52 ± 1.04 Cb	27.30 ± 1.04 Cbc	27.82 ± 1.04 Bc
	Black	27.57 ± 1.82 Aa	29.64 ± 1.30 Ab	29.12 ± 1.56 ABb	29.12 ± 1.56 Ab
	Pink	26.26 ± 0.78 ABa	27.82 ± 1.30 BCb	28.08 ± 1.56 BCb	27.82 ± 1.04 Bb

Different lowercase letters in row show a statistical difference (*p* < 0.05, Tukey). Different uppercase letters in column show a statistical difference (for each silicone individually) (*p* < 0.05, Tukey).

**Table 2:** Mean (N/mm) ± standard deviation for the tear strength test of the A-2186 and MDX4-4210 silicones, according to the period and pigmentation (or no pigmentation).

Silicone	Pigmentation	Period			
		Initial	252 hours	504 hours	1008 hours
A-2186	Without pigmentation	35.67 ± 5.97 Aa	44.88 ± 7.74 ABb	32.73 ± 4.80 ABa	42.53 ± 9.50 Ab
	Bronze	37.92 ± 4.70 Aa	48.70 ± 3.92 Ab	35.28 ± 6.27 Aac	32.63 ± 8.03 Bc
	Black	36.65 ± 4.21 Aa	42.04 ± 3.92 Bb	31.55 ± 2.64 ABc	38.90 ± 6.37 Aab
	Pink	37.24 ± 3.72 Ab	42.04 ± 5.58 Ba	31.45 ± 4.11 Bc	38.12 ± 10.38 ABab

MDX4-4210	Without pigmentation	13.72 ± 1.47 Ab	23.71 ± 11.56 Aa	21.26 ± 7.44 Aa	22.73 ± 7.54Aa
	Bronze	14.99 ± 0.88 Aa	18.42 ± 2.84 Ba	14.40 ± 1.37 Ba	18.42 ± 3.43 ABa
	Black	15.09 ± 1.07 Aa	17.54 ± 0.88 Ba	15.48 ± 0.98 Ba	20.09 ± 3.03 ABa
	Pink	15.97 ± 1.37 Aa	16.95 ± 1.86 Ba	14.60 ± 1.07 Ba	15.68 ± 0.88 Ba

Different lowercase letters in row show a statistical difference (*p* < 0.05, Tukey). Different uppercase letters in column show a statistical difference (for each silicone individually) (*p* < 0.05, Tukey).

**Table 3:** Mean (Shore A units) ± standard deviation (SD) for the hardness test of the MDX4-4210 and A-2186 silicones, comparing these silicones based on the same period and pigmentation (or no pigmentation).

Pigmentation	Period	Silicone	Mean ± SD	*p* value
Without pigmentation	Initial	A-2186	29.64 ± 1.56	*p* < 0.05^*∗*^
		MDX4-4210	27.30 ± 1.82	
	252 hours	A-2186	31.46 ± 1.56	*p* < 0.05^*∗*^
		MDX4-4210	28.34 ± 2.08	
	504 hours	A-2186	31.46 ± 0.78	*p* < 0.05^*∗*^
		MDX4-4210	29.64 ± 1.30	
	1008 hours	A-2186	30.16 ± 2.60	*p* > 0.05
		MDX4-4210	30.16 ± 1.04	

Bronze	Initial	A-2186	29.64 ± 2.34	*p* < 0.05^*∗*^
		MDX4-4210	25.48 ± 1.04	
	252 hours	A-2186	30.94 ± 1.56	*p* < 0.05^*∗*^
		MDX4-4210	26.52 ± 1.04	
	504 hours	A-2186	31.72 ± 1.04	*p* < 0.05^*∗*^
		MDX4-4210	27.30 ± 1.04	
	1008 hours	A-2186	30.94 ± 0.26	*p* < 0.05^*∗*^
		MDX4-4210	27.82 ± 1.04	

Black	Initial	A-2186	30.94 ± 1.04	*p* < 0.05^*∗*^
		MDX4-4210	27.57 ± 1.82	
	252 hours	A-2186	32.76 ± 1.04	*p* < 0.05^*∗*^
		MDX4-4210	29.64 ± 1.30	
	504 hours	A-2186	31.98 ± 1.30	*p* < 0.05^*∗*^
		MDX4-4210	29.12 ± 1.56	
	1008 hours	A-2186	33.54 ± 1.30	*p* < 0.05^*∗*^
		MDX4-4210	29.12 ± 1.56	

Pink	Initial	A-2186	28.08 ± 2.08	*p* < 0.05^*∗*^
		MDX4-4210	26.26 ± 0.78	
	252 hours	A-2186	31.20 ± 1.30	*p* < 0.05^*∗*^
		MDX4-4210	27.82 ± 1.30	
	504 hours	A-2186	31.98 ± 1.04	*p* < 0.05^*∗*^
		MDX4-4210	28.08 ± 1.56	
	1008 hours	A-2186	32.24 ± 0.78	*p* < 0.05^*∗*^
		MDX4-4210	27.82 ± 1.04	

^*∗*^Significant statistical difference (*p* < 0.05, Tukey).

**Table 4:** Mean (N/mm) ± standard deviation (SD) for the tear strength test of the MDX4-4210 and A-2186 silicones, comparing these silicones based on the same period and pigmentation (or no pigmentation).

Pigmentation	Period	Silicone	Mean ± SD	*p* value
Without pigmentation	Initial	A-2186	35.67 ± 5.97	*p* < 0.05^*∗*^
		MDX4-4210	13.72 ± 1.47	
	252 hours	A-2186	44.88 ± 7.74	*p* < 0.05^*∗*^
		MDX4-4210	23.71 ± 11.56	
	504 hours	A-2186	32.73 ± 4.80	*p* < 0.05^*∗*^
		MDX4-4210	21.26 ± 7.44	
	1008 hours	A-2186	42.53 ± 9.50	*p* < 0.05^*∗*^
		MDX4-4210	22.73 ± 7.54	

Bronze	Initial	A-2186	37.92 ± 4.70	*p* < 0.05^*∗*^
		MDX4-4210	14.99 ± 0.88	
	252 hours	A-2186	48.70 ± 3.92	*p* < 0.05^*∗*^
		MDX4-4210	18.42 ± 2.84	
	504 hours	A-2186	35.28 ± 6.27	*p* < 0.05^*∗*^
		MDX4-4210	14.40 ± 1.37	
	1008 hours	A-2186	32.63 ± 8.03	*p* < 0.05^*∗*^
		MDX4-4210	18.42 ± 3.43	

Black	Initial	A-2186	36.65 ± 4.21	*p* < 0.05^*∗*^
		MDX4-4210	15.09 ± 1.07	
	252 hours	A-2186	42.04 ± 3.92	*p* < 0.05^*∗*^
		MDX4-4210	17.54 ± 0.88	
	504 hours	A-2186	31.55 ± 2.64	*p* < 0.05^*∗*^
		MDX4-4210	15.48 ± 0.98	
	1008 hours	A-2186	38.90 ± 6.37	*p* < 0.05^*∗*^
		MDX4-4210	20.09 ± 3.03	

Pink	Initial	A-2186	37.24 ± 3.72	*p* < 0.05^*∗*^
		MDX4-4210	15.97 ± 1.37	
	252 hours	A-2186	42.04 ± 5.58	*p* < 0.05^*∗*^
		MDX4-4210	16.95 ± 1.86	
	504 hours	A-2186	31.45 ± 4.11	*p* < 0.05^*∗*^
		MDX4-4210	14.60 ± 1.07	
	1008 hours	A-2186	38.12 ± 10.38	*p* < 0.05^*∗*^
		MDX4-4210	15.68 ± 0.88	

^*∗*^Significant statistical difference (*p* < 0.05, Tukey).

## Data Availability

The data used to support the findings of this study are included within the article.
